# Real-time monitoring of formaldehyde exposure and acute health symptoms in healthcare workers in Morocco: a cross-sectional study

**DOI:** 10.1590/0102-311XEN191325

**Published:** 2026-08-21

**Authors:** Loubna Sadki, Chadia Ouazzani, Abdallah Dami, Lhousaine Balouch, Abdellah Moustaghfir

**Affiliations:** 1 Faculty of Medicine and Pharmacy, Mohammed V University, Rabat, Morocco.; 2 Faculty of Dental Medicine, Mohammed V University, Rabat, Morocco.

**Keywords:** Formaldehyde, Toxicity, Occupational Exposure, Formaldeído, Toxicidade, Exposição Ocupacional, Formaldehído, Toxicidad, Exposición Profesional

## Abstract

Formaldehyde remains widely used in clinical and dental procedures, particularly in low- and middle-income countries with limited regulatory oversight, where occupational exposure data remain scarce. This cross-sectional study evaluated real-time formaldehyde exposure and its association with acute health symptoms among 180 healthcare workers in Morocco. Airborne formaldehyde was measured across 12 hospital departments during eight-hour shifts using a calibrated semiconductor dosimeter, alongside a structured symptom questionnaire. Associations were assessed using multivariate logistic regression and Spearman’s correlation. All mean concentrations remained below the Moroccan occupational limit of 0.25mg/m^3^ (range: 0.048-0.196mg/m^3^; 19-78% of the limit). Nevertheless, highly exposed workers reported significantly more symptoms, including eye irritation (72.5%), headaches (63%), and respiratory discomfort (56.5%). Prolonged exposure (≥ 8 hours/day, ≥ 3 days/week) independently predicted multiple symptoms (AOR = 3.2; 95%CI: 1.7-5.9), as did inadequate ventilation (AOR = 2.4) and inconsistent PPE use (AOR = 1.9). Years of exposure correlated moderately with symptom count (ρ = 0.41; p < 0.01). Despite the cross-sectional design, formaldehyde exposure was strongly associated with acute symptoms even at sub-threshold concentrations, suggesting current limits may not fully protect workers. Strengthening preventive measures is essential in resource-limited settings. These findings support integrated exposure-surveillance approaches in low- and middle-income countries. Longitudinal studies with biomarkers are needed to refine causal inference and inform policy.

## Resumo

O formaldeído continua amplamente utilizado em procedimentos clínicos e odontológicos, sobretudo em países de renda baixa e média com supervisão regulatória limitada, onde faltam dados sobre exposição ocupacional. Este estudo transversal avaliou a exposição ao formaldeído em tempo real e sua associação com sintomas agudos em 180 profissionais de saúde no Marrocos. O formaldeído no ar foi medido em 12 departamentos hospitalares durante turnos de oito horas com dosímetro semicondutor calibrado, junto a um questionário estruturado de sintomas. As associações foram avaliadas por regressão logística multivariada e correlação de Spearman. Todas as concentrações médias ficaram abaixo do limite marroquino de 0,25mg/m^3^ (variação: 0,048-0,196mg/m^3^; 19-78% do limite). Ainda assim, trabalhadores altamente expostos relataram significativamente mais sintomas, incluindo irritação ocular (72,5%), cefaleia (63%) e desconforto respiratório (56,5%). A exposição prolongada (≥ 8 horas/dia, ≥ 3 dias/semana) previu independentemente múltiplos sintomas (OR ajustado = 3,2; IC95%: 1,7-5,9), assim como ventilação inadequada (OR ajustado = 2,4) e uso inconsistente de EPI (OR ajustado = 1,9). Os anos de exposição correlacionaram-se moderadamente com o número de sintomas (ρ = 0,41; p < 0,01). Apesar do desenho transversal, a exposição ao formaldeído esteve fortemente associada a sintomas agudos mesmo em concentrações sublimiares, sugerindo que os limites atuais podem não proteger totalmente os trabalhadores. Fortalecer medidas preventivas é essencial em contextos de recursos limitados. Esses achados apoiam abordagens integradas de vigilância da exposição em países de renda baixa e média. Estudos longitudinais com biomarcadores são necessários para refinar a inferência causal e orientar políticas.

## Resumen

El formaldehído sigue muy utilizado en procedimientos clínicos y dentales, sobre todo en países de ingresos bajos y medios con supervisión regulatoria limitada, donde faltan datos sobre exposición ocupacional. Este estudio transversal evaluó la exposición al formaldehído en tiempo real y su asociación con síntomas agudos en 180 profesionales de salud marroquíes. El formaldehído en el aire se midió en 12 departamentos hospitalarios durante turnos de ocho horas con un dosímetro semiconductor calibrado, junto con un cuestionario estructurado de síntomas. Las asociaciones se evaluaron mediante regresión logística multivariada y correlación de Spearman. Todas las concentraciones medias se mantuvieron por debajo del límite marroquí de 0,25mg/m^3^ (rango: 0,048-0,196mg/m^3^; 19-78% del límite). Sin embargo, los trabajadores muy expuestos presentaron significativamente más síntomas, como irritación ocular (72,5%), cefalea (63%) y dificultad respiratoria (56,5%). La exposición prolongada (≥ 8 horas/día, ≥ 3 días/semana) predijo independientemente múltiples síntomas (OR ajustado = 3,2; IC95%: 1,7-5,9), al igual que la ventilación inadecuada (OR ajustado = 2,4) y el uso inconsistente de EPP (OR ajustado = 1,9). Los años de exposición se correlacionaron moderadamente con el número de síntomas (ρ = 0,41; p < 0,01). Pese al diseño transversal, la exposición al formaldehído estuvo fuertemente asociada con síntomas agudos incluso en concentraciones subumbrales, lo que sugiere que los límites actuales podrían no proteger del todo a los trabajadores. Fortalecer medidas preventivas es esencial en entornos de recursos limitados. Estos hallazgos respaldan enfoques integrados de vigilancia de la exposición en países de ingresos bajos y medios. Se necesitan estudios longitudinales con biomarcadores para refinar la inferencia causal y orientar políticas.

## Introduction

In countless healthcare environments worldwide, the faint scent of formaldehyde often goes unnoticed − an imperceptible yet persistent presence embedded in the daily routines of clinical and dental practice. Valued for its potent antimicrobial and tissue-preserving properties, formaldehyde continues to serve as a mainstay in procedures ranging from specimen fixation to the sterilization of medical instruments. However, its widespread usage obscures a pressing concern: formaldehyde is a group 1 human carcinogen according to the International Agency for Research on Cancer (IARC) ^1^ and is increasingly associated with respiratory irritation, genotoxic effects, and hematologic malignancies ^2^ ^,^ ^3^.

While occupational exposure standards and risk mitigation strategies are well established in high-income countries, systematic monitoring and protective infrastructures remain less consistently implemented in many low- and middle-income countries (LMICs), often due to structural or resource-related constraints. In regions such as North Africa, healthcare professionals working in high-exposure areas − such as pathology laboratories, operating rooms, and dental clinics − may face unquantified levels of formaldehyde exposure in the absence of routine air sampling and health surveillance protocols ^4^. The recent literature highlights the cumulative and synergistic effects of repeated low-dose exposure, which may progressively contribute to chronic health outcomes in healthcare professionals ^5^.

To address this critical gap in the data and occupational health surveillance, we conducted a two-phase field investigation in 12 clinical and laboratory units across the Al Hoceima Province, Morocco. The first phase involved real-time formaldehyde monitoring using a TiO_2_-based semiconductor dosimeter (JMS11), benchmarked against national and international exposure limits ^6^ ^,^ ^7^ ^,^ ^8^. The second phase comprised a structured clinical assessment in which healthcare professionals reported the presence and severity of acute symptoms plausibly linked to formaldehyde exposure, such as mucosal irritation, headaches, and respiratory discomfort ^9^.

To our knowledge, this is the first study in North Africa to implement real-time formaldehyde monitoring and structured clinical symptom surveillance in healthcare workers. While similar approaches have occurred in high-income settings, their use in LMICs remains exceptionally rare. This dual-method design addresses the environmental dimension of chemical exposure and integrates the clinical manifestations of that exposure in the same occupational context, thus offering a novel and scalable model for chemical risk surveillance in resource-limited environments.

## Methodology

A dual methodological approach was implemented to rigorously assess occupational formaldehyde exposure and its potential acute health effects in healthcare professionals: (i) real-time environmental monitoring using a semiconductor-based sensor, and (ii) evaluation of self-reported symptoms by a structured and validated questionnaire. This integrated design enabled objectively quantified airborne formaldehyde levels and subjectively assessed associated health manifestations in clinical and dental settings.

### Real-time monitoring of formaldehyde exposure

#### Sensor description

A compact (133 × 43 × 24 mm) device (Real Technology Co. Ltd., https://www.realtechng.com/) equipped with an LED display and a TiO_2_-based semiconductor sensitive to total volatile organic compounds (TVOCs), (including formaldehyde) operates by detecting changes in conductivity induced by gas molecule adsorption, providing immediate readings in mg/m^3^ ^10^.

While passive dosimetry remains the gold standard for cumulative exposure assessment, its reliance on post-sampling laboratory analysis offers logistical limitations in field settings. Accordingly, the JMS11 was selected due to its ISO 17025-compliant performance, proven reliability in healthcare environments ^11^, and its ability to capture short-term concentration variability in real time.

Although the JMS11 primarily responds to TVOCs rather than providing chemically selective formaldehyde measurements, similar low-cost semiconductor-based devices have been used in applied indoor air quality studies as field-adapted tools to characterize relative volatile organic compounds exposure patterns when reference methods such as 2,4-Dinitrophenylhydrazine (DNPH) cartridge sampling or thermal desorption gas chromatography mass spectrometry (TD-GC-MS) are operationally unfeasible ^12^ ^,^ ^13^. In environments that intentionally and routinely use formaldehyde for clinical purposes, TVOC measurements were considered a practical proxy to assess comparative exposures between units even if absolute concentrations may be influenced by co-existing volatile compounds.

#### Study sites and sampling design

In this study, 12 clinical and laboratory units were selected to capture a broad spectrum of formaldehyde application contexts. These included biochemistry, hematology, parasitology departments, surgical unit, dental clinics, sterilization units, endoscopy rooms, pharmaceutical compounding areas, and microbiological laboratories for water and food analysis. Each unit was continuously monitored during normal working hours in Morocco from 8:30 AM to 4:30 PM. Formaldehyde concentrations were measured hourly throughout this period, collecting time-resolved exposure data. These hourly measurements were then averaged over the eight-hour work shift to calculate the mean daily exposure level for each unit.

#### Measurement protocol

During each eight-hour work shift (8:30 AM to 4:30 PM), formaldehyde concentrations were measured hourly using the JMS11 semiconductor-based dosimeter. All measurements were performed by trained members of the research team, who were responsible for device handling, calibration verification, and data recording. Hospital staff did not operate the device. Prior to field deployment, the research team received standardized training on the JMS11 sensor to ensure consistency and methodological reliability across all study sites. To minimize operator-dependent variability, the same standardized measurement protocol was applied across all units.

The device was positioned near workers’ breathing zone, typically within 0.5 meters of active work surfaces such as laboratory benches, dental chairs, or specimen handling areas to better reflect actual personal exposure levels. Unlike continuous automatic sampling systems, the JMS11 operates via manual activation: the researcher initiates each measurement by pressing its on/off button. The device then provides discrete, portable “snapshot” readings analogous to a handheld thermometer. In total, eight consecutive hourly measurements were collected per session and then averaged to obtain the mean exposure level per shift.

#### Environmental conditions

All measurements were carried out in clinical laboratories and hospital units equipped with environmental control systems that continuously display ambient conditions. Throughout the monitoring period, the temperature was consistently maintained at 22°C and the atmospheric pressure at 1 bar, as indicated on the internal displays of each unit. These areas were equipped with mechanical ventilation systems (standard heating, ventilation, and air conditioning HVAC systems or air extraction systems), ensuring consistent air renewal in line with hospital hygiene standards. Access to these units was restricted to authorized personnel only, minimizing external interference and ensuring the reliability of formaldehyde measurements.

#### Quality assurance and data processing

The following quality control procedures were implemented to ensure result accuracy and reproducibility:

i) Calibration and Blanks: all JMS11 units were factory-calibrated and further verified using certified formaldehyde gas standards in accordance with ISO 17025:2017 ^11^ protocols prior to deployment ^14^. Daily blank measurements were conducted in a clean-air environment to validate sensor stability ^15^ with the recommended regular zero measurements for baseline correction.

ii) Time-Weighted Averages (TWA): Collected hourly data were transcribed into spreadsheets and used to calculate eight-hour time-weighted averages (TWA 8h) in accordance with occupational exposure guidelines ^6^ ^,^ ^7^.

iii) Regulatory Benchmarking: The TWA8h of each unit was evaluated against the Moroccan occupational exposure limit (OEL, 8 hours) for formaldehyde (set at 0.25mg/m^3^) ^8^. Units were classified as either “compliant” or “non-compliant” based on this threshold.

### Acute symptom assessment via structured questionnaire

#### Data collection: questionnaire for healthcare professionals

A tailored questionnaire was developed to systematically capture data on the acute symptoms healthcare professionals exposed to formaldehyde in clinical settings experience. Data collection followed a structured, multi-phase approach to ensure reliability and comprehensiveness.

#### a) Design and preliminary validation

The questionnaire was based on validated international models, including the Occupational Safety and Health Assessment questionnaires − used for evaluating health effects of chemical exposure ^16^. It was adapted to Moroccan healthcare and structured into three key sections:

Section 1: demographic and professional characteristics (age, gender, role, years of experience, exposure frequency).

Section 2: clinical symptoms (ocular, respiratory, dermal, systemic manifestations).

Section 3: environmental and occupational determinants, including ventilation, personal protective equipment (PPE) use, and types of procedures.

To ensure clarity and cultural appropriateness, a pilot test was conducted with 10 healthcare professionals. Refinements were made based on their feedback ^17^. A panel of occupational health experts reviewed the content to confirm validity.

Sample size was calculated on G*Power (http://www.psycho.uni-duesseldorf.de/abteilungen/aap/gpower3), assuming a 95% confidence interval (95%CI) and 80% power, indicating a minimum of 180 participants ^18^. In total, 220 healthcare professionals completed the final questionnaire.

A non-probabilistic convenience sampling approach was used, targeting all eligible healthcare professionals in the selected units during the study period. The number of 220 participants reflects the total number of professionals who agreed to participate among those approached, exceeding the minimum required sample size of 180 to account for potential exclusions and incomplete responses.

To ensure response relevance and the accurate classification of exposure, the questionnaire was only administered to healthcare professionals working within 12 clinical and laboratory units that had been identified as potentially using or storing formaldehyde. These units were selected based on prior environmental assessments and internal facility reports that confirmed the presence or potential use of formaldehyde-containing products. This targeted distribution strategy was designed to ensure that only professionals with plausible exposure scenarios were surveyed, improving the specificity of the exposure assessment and enhancing its internal validity.

#### b) Inclusion and exclusion criteria

Eligible participants were healthcare professionals (physicians, nurses, laboratory technicians, dentists...) working in selected clinical and laboratory units during the study period. Exposure frequency and duration were assessed via structured questionnaire. High exposure was defined a priori as ≥ 8 hours/day and ≥ 3 days/week of formaldehyde-related activities in routinely handling units. Professionals with chronic conditions potentially confounding symptom reporting, incomplete questionnaires, or refusals were excluded. All others, including staff from departments without documented formaldehyde use, were classified as minimally/non-exposed.

#### c) Questionnaire administration

To minimize selection bias, a mixed-mode distribution strategy was used. The questionnaire was shared electronically (email and Google Forms, https://docs.google.com/forms) and in paper format in institutions with limited internet access. Participants were informed of the study objectives and data protection measures. Completion of the questionnaire was considered as implicit consent, in accordance with accepted digital research ethics ^19^.

Importantly, participants had no access to the results of environmental formaldehyde measurements at the time of questionnaire completion. Air monitoring and symptom assessment were conducted independently, and environmental measurement results were disclosed only after were collection. This methodological separation was implemented to minimize potential reporting bias related to awareness of workplace formaldehyde levels.

#### d) Data collection and management

Data were collected over three months. A double data entry protocol was implemented using KoBoToolbox (https://www.kobotoolbox.org), a free and open-source electronic data capture system) to reduce input errors.

Environmental concentrations in each unit were evaluated against the Moroccan occupational exposure limit (OEL, 0.25mg/m^3^) for regulatory benchmarking. This threshold was used to assess compliance but not for individual exposure stratification.

This indirect method has been validated in previous occupational exposure studies ^20^ ^,^ ^21^.

#### e) Statistical analysis

For analytical purposes, participants were classified into exposure groups based on (i) professional assignment to units with documented formaldehyde use and (ii) reported occupational exposure frequency (≥ 8 hours/day and ≥ 3 days/week). Participants meeting both criteria were classified as highly exposed. All others − including staff from departments without documented formaldehyde use or those who failed to meet the predefined exposure threshold − were categorized as minimally/non-exposed.

The designation “minimally/non-exposed” was adopted to reflect that, although no documented formaldehyde use or routine handling was identified in those departments, complete absence of exposure was unable to be definitively confirmed.

Descriptive analyses (means, standard deviations, and frequencies) were conducted on Jamovi (The Jamovi Project, https://www.jamovi.org/). Differences in symptom prevalence by exposure category were assessed by the chi-squared and independent t-tests.

A multivariate logistic regression model was applied to examine the association between formaldehyde exposure and reported symptoms, adjusting for potential confounders (including age, smoking status, and PPE use).

To support causal interpretation, a comparison group of 60 non-exposed healthcare professionals was recruited from departments with no documented formaldehyde use or storage (based on institutional inventory records and confirmation by unit supervisors). These departments were physically separated from high-exposure areas and performed no procedures involving formaldehyde-containing products. Participants were selected using the same convenience-based strategy among eligible staff in the study period. The size of the comparison group was determined based on staff availability in these departments.

However, the comparison group was not formally frequency-matched by age, gender, or professional role, which represents a minor methodological limitation.

### Ethical and administrative approval

Prior to the initiation of this study, formal administrative approval (Reference n. 7314, dated 25 December 2024) was granted by the Regional Directorate of Health and Social Protection of the Tangier-Tetouan-Al Hoceima Region, Morocco, authorizing the conduct of this study in healthcare facilities under the Provincial Delegation of the Ministry of Health and Social Protection of Al Hoceima.

In parallel, the symptom-based questionnaire component was conducted independently. In line with international ethical research standards, participants were informed of the objectives of this study, their voluntary involvement, and the measures to ensure data confidentiality. Completion of the questionnaire was regarded as implicit informed consent, consistent with accepted practices for anonymized, minimal-risk research involving human subjects. To ensure confidentiality, no personally identifiable information or institutional affiliations were included in the final dataset or publications.

## Results

### Results of real-time formaldehyde monitoring (TWA8h)

The highest mean TWA8h formaldehyde concentration occurred in the surgical unit (0.196mg/m^3^; 78% of the OEL), followed by the dental surgery room (0.187mg/m^3^; 75% of the OEL) and the dental material preparation unit (0.180mg/m^3^; 72% of the OEL). The lowest mean concentration occurred in the hematology laboratory (0.048mg/m^3^; 19% of the OEL) (Table 1).

**Table 1:** Eight-hour TWA formaldehyde concentrations and compliance by clinical unit.

Service/Unit	Hourly values (mg/m³)	Mean (mg/m³)	% of OEL	Compliance
Surgical unit	0.33 - 0.35 - 0.36 - 0.34 - 0.31 - 0.32 - 0.30 - 0.34	0.196	78%	Yes
Dental surgery room	0.17 - 0.18 - 0.20 - 0.22 - 0.21 - 0.19 - 0.17 - 0.16	0.187	75%	Yes
Dental materials preparation	0.25 - 0.25 - 0.24 - 0.23 - 0.24 - 0.25 - 0.23 - 0.24	0.180	72%	Yes
Parasitology laboratory	0.14 - 0.15 - 0.16 - 0.17 - 0.16 - 0.15 - 0.13 - 0.12	0.148	59%	Yes
Pharmaceutical preparation Area	0.12 - 0.14 - 0.15 - 0.16 - 0.15 - 0.14 - 0.13 - 0.12	0.138	55%	Yes
Dental treatment rooms	0.11 - 0.13 - 0.14 - 0.15 - 0.13 - 0.12 - 0.11 - 0.10	0.123	49%	Yes
Food microbiology Laboratory	0.09 - 0.11 - 0.12 - 0.13 - 0.12 - 0.11 - 0.10 - 0.09	0.108	43%	Yes
Biochemistry laboratory	0.08 - 0.10 - 0.12 - 0.11 - 0.10 - 0.09 - 0.08 - 0.07	0.093	37%	Yes
Water microbiology Laboratory	0.06 - 0.07 - 0.08 - 0.08 - 0.08 - 0.07 - 0.06 - 0.06	0.070	28%	Yes
Endoscopy room	0.06 - 0.07 - 0.08 - 0.09 - 0.08 - 0.07 - 0.06 - 0.05	0.070	28%	Yes
Sterilization room	0.05 - 0.06 - 0.07 - 0.08 - 0.07 - 0.06 - 0.05 - 0.04	0.060	24%	Yes
Hematology laboratory	0.05 - 0.06 - 0.06 - 0.06 - 0.05 - 0.04 - 0.04 - 0.03	0.048	19%	Yes

Note: OEL: Moroccan occupational exposure limit (0.25mg/m^3^, TWA8h). For comparison, the American Conference of Governmental Industrial Hygienists recommends a ceiling Threshold Limit Value (TLV-C) of 0.3ppm (~0.37mg/m³) for formaldehyde. The Moroccan standard does not currently specify a short-term exposure limit (STEL) or ceiling value.

Hourly concentrations across all units ranged from a minimum of 0.03mg/m^3^ (hematology) to a maximum of 0.24mg/m^3^. The surgical unit showed the highest short-term exposure during peak activity. This study obtained a modest within-unit variability (difference between highest and lowest hourly reading), averaging 0.07mg/m³, indicating relatively stable formaldehyde levels over each monitoring period.

No unit exceeded the 0.25mg/m^3^ threshold. Thus, this study deemed all sites as compliant with national occupational exposure limits. These findings show that, under current operational conditions and engineering controls, formaldehyde exposure remains within acceptable bounds across diverse medical and dental setting.

### Results and analyses of the clinical questionnaire

#### Data cleaning and sample description

Of the 220 questionnaires distributed, the research team received 199 completed ones. After applying the predefined exclusion criteria − including incomplete responses and chronic conditions potentially confounding symptom reporting −, this study excluded 19 questionnaires. The final analytical sample comprised 180 participants.

In accordance with the predefined exposure criteria, this research classified 74 participants as highly exposed (≥ 8 hours/day and ≥ 3 days/week of formaldehyde-related activities in routinely handling units), categorizing the remaining 106 participants as minimally/non-exposed, including 60 staff members from departments without documented formaldehyde use and 46 personnel from formaldehyde-handling units who failed to meet the high-exposure threshold.

#### Symptom prevalence by exposure status

Of the 180 valid responses retained for analysis, the most commonly reported symptoms potentially associated with formaldehyde exposure were eye irritation (72.5%), headaches (63%), respiratory discomfort (56.5%), excessive fatigue (47.1%), nausea (38.4%), and dizziness (37%).

**Figure 1:**
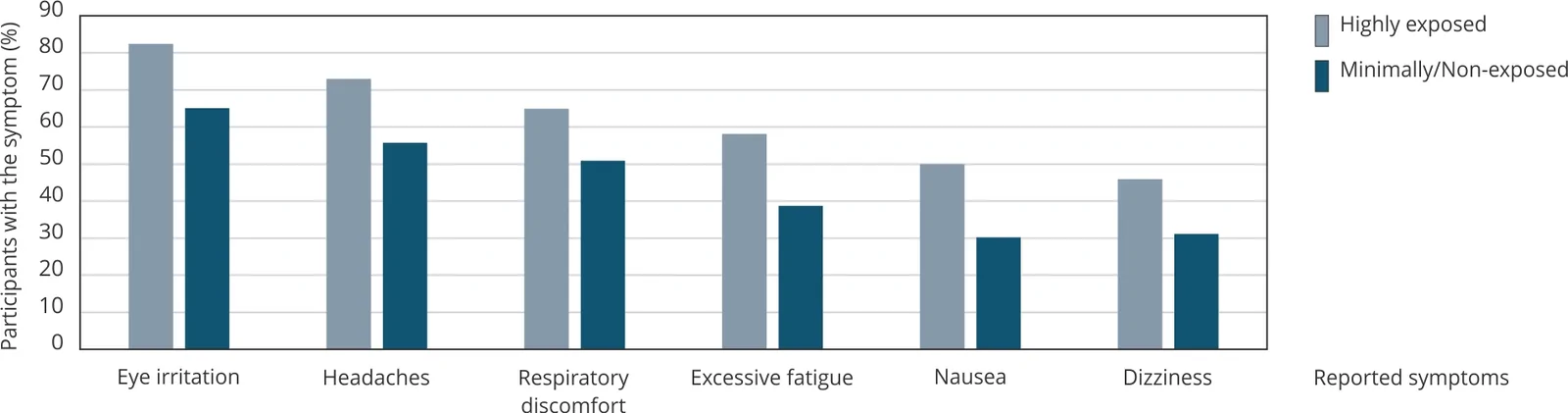
Prevalence of self-reported acute symptoms according to formaldehyde exposure.

Note: prevalence of self-reported acute symptoms in healthcare workers classified as highly exposed (n = 74) and minimally/non-exposed (n = 106) according to predefined exposure criteria. Percentages represent the proportion of participants reporting each symptom. Statistical comparisons were performed using the chi-squared test (see Table 2).

When stratified by exposure group, these symptoms showed a marked greater prevalence in highly exposed participants (n = 74) than in minimally or non-exposed ones (n = 106). Mucosal irritation symptoms (such as eye and respiratory tract discomfort) and general systemic complaints (fatigue, dizziness) showed a particularly pronounced prevalence difference. Figure 1 visually summarizes these differences.

The chi-squared tests confirmed statistically significant associations between high exposure status and each of the six reported symptoms (all p-values < 0.05), supporting the hypothesis of a link between occupational formaldehyde exposure and the development of acute health complaints.

These results highlight the importance of assessing workplace exposure in clinical and dental environments where formaldehyde is used.

Table 2 compares symptom prevalence by exposure group in detail.

**Table 2:** Symptom prevalence by exposure group.

Symptom	Highly exposed ENT#091;n = 74ENT#093; n (%)	Minimally/Non-exposed ENT#091;n = 106ENT#093; n (%)	χ²	p-value
Eye irritation	61 (82.4)	69 (65.1)	6.55	0.010
Headaches	54 (73.0)	59 (55.7)	5.52	0.019
Respiratory discomfort	48 (64.9)	54 (50.9)	3.76	0.052
Excessive fatigue	43 (58.1)	41 (38.7)	6.98	0.008
Nausea	37 (50.0)	32 (30.2)	8.10	0.004
Dizziness	34 (45.9)	33 (31.1)	4.44	0.035

χ²: chi-square test.

#### Predictors of symptom clustering (≥ 2 symptoms)

This study created a binary outcome variable indicating the presence of two or more reported symptoms to explore the factors associated with a higher symptom burden. It performed a multivariate logistic regression analysis, adjusting it for potential confounding variables (including age, gender, PPE use, and ventilation adequacy).

Results indicated that prolonged occupational exposure to formaldehyde (≥ 8 hours/day, ≥ 3 days/week) constituted the most significant independent predictor of symptom clustering, with an adjusted odds ratio (AOR) of 3.2 (95%CI: 1.7-5.9; p < 0.001). Inadequate workplace ventilation (AOR = 2.4; 95%CI: 1.3-4.5; p = 0.004) and irregular or inconsistent PPE use (AOR = 1.9; 95%CI: 1.1-3.3; p = 0.022) were also significantly associated with increased odds of reporting multiple symptoms.

Age and professional category (e.g., technician vs. assistant) showed no statistical significance in the adjusted model. These findings emphasize the critical role of environmental controls and protective practices in mitigating symptom burden (Table 3).

**Table 3:** Multivariate logistic regression for ≥ 2 symptoms.

Parameter	AOR	95%CI	p-value
Prolonged exposure (≥ 8 hours/day, 3 days/week)	3.20	1.70-5.90	< 0.001
Inadequate ventilation	2.40	1.30-4.50	0.004
Irregular PPE use	1.90	1.10-3.30	0.022
Age (per year)	1.01	0.98-1.04	0.442
Gender (female vs. male)	1.15	0.70-1.90	0.575
Profession (technician vs. other)	1.12	0.60-2.10	0.683

95%CI: 95% confidence interval; AOR: adjusted odds ratio; PPE: personal protective equipment.

#### Cumulative effects of exposure duration

A statistically significant moderate positive correlation occurred between the number of years of occupational exposure to formaldehyde and the total number of symptoms reported by participants (Spearman’s ρ = 0.41; p < 0.01). This correlation suggests a potential cumulative dose-response relationship, whereby long-term low-level exposure may progressively increase symptom burden.

**Figure 2:**
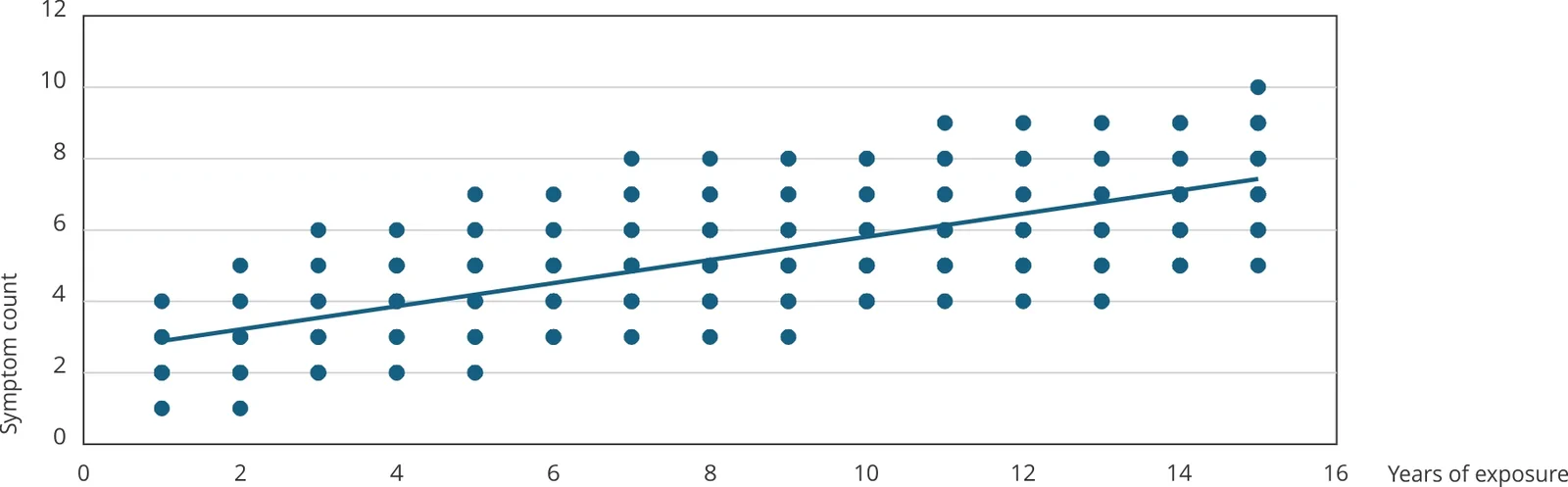
Correlation between years of occupational formaldehyde and total number of reported symptoms.

Note: scatter plot illustrating the association between years of occupational formaldehyde exposure and total number of self-reported acute symptoms among healthcare workers (n = 180). Each point represents one participant. The solid line indicates the linear trend for visualization purposes. A moderate positive correlation was observed (Spearman’s ρ = 0.41; p < 0.01).

These findings align themselves with the literature on the chronic health effects of formaldehyde and underscore the importance of implementing preventive measures early in workers’ careers. The relationship between exposure duration and symptom count (Figure 2) suggests an association consistent with a dose-response pattern.

## Discussion

This study provides one of the first comprehensive assessments of formaldehyde exposure and related acute symptoms in healthcare workers in Moroccan clinical and dental settings. By combining environmental measurements and structured self-report questionnaires, our findings offer robust evidence for a significant association − though not necessarily a causal link − between occupational formaldehyde exposure and the prevalence of acute mucosal and systemic symptoms.

### Formaldehyde exposure levels across clinical units

All 122 monitored clinical units reported TWA8h formaldehyde concentrations below the 0.25mg/m^3^ national OEL. The highest mean concentrations occurred in the surgical unit (0.196mg/m^3^), followed by the dental surgery room (0.187mg/m^3^) and the dental material preparation area (0.180mg/m^3^). While these values comply with existing standards, they still represent relatively intense exposure scenarios that may involve cumulative health risks, particularly in the absence of adequate engineering controls.

Despite no regulatory exceedances, evidence from international studies highlights that adverse effects can occur even at sub-OEL concentrations, particularly among sensitized individuals or those with prolonged contact ^22^ ^,^ ^23^. Khoshakhlagh et al. ^3^ reported similar sub-threshold levels in Iranian hospital laboratories, yet observed a significant correlation with upper respiratory symptoms ^24^. These findings confirm that clinical and dental environments remain key risk areas for low-dose chronic exposure ^6^.

The Moroccan OEL for formaldehyde (0.25mg/m^3^, TWA8h) is broadly comparable to international benchmarks ^8^. For example, the American Conference of Governmental Industrial Hygienists recommends a 0.3ppm (~0.37mg/m^3^) threshold limit value, indicating that concentrations should always remain below it ^25^. Importantly, occupational exposure limits are regulatory risk-management tools rather than biological thresholds for adverse health effects, as emphasized by international expert committees ^26^.

Since the IARC classifies formaldehyde as a Group 1 human carcinogen ^1^, sole reliance on occupational exposure limits represents an inherent limitation. For carcinogenic agents, OELs function as regulatory benchmarks rather than indicators of a biologically safe threshold, possibly failing to fully account for cumulative or probabilistic cancer risk associated with long-term exposure. Consequently, sole reliance on OEL compliance may underestimate potential health risks, especially in settings with repeated low-dose exposure or short-term concentration peaks.

### Symptom prevalence and occupational correlates

The questionnaire data showed a high prevalence of symptoms in healthcare workers, including eye irritation (72.5%), headaches (63%), respiratory discomfort (56.5%), and dizziness (37%). These findings are consistent with recent occupational health studies. For instance, a cross-sectional study conducted at Wollo University in Ethiopia ^27^ reported a high prevalence of acute symptoms among medical students and anatomy staff exposed to formaldehyde vapors during cadaver dissection, including unpleasant odor perception (72.7%), tiredness/dizziness (44.3%), and eye watering (39.8%). Similarly, Fan et al. ^5^ found a strong association between low-dose formaldehyde exposure and allergic manifestations in China, including skin irritation, allergic rhinitis, and dermatitis. The stratified analysis in our study further confirmed a statistically significant relationship between higher exposure levels and reported symptom frequency.

These symptoms are aligned with the irritative and neurotoxic effects of formaldehyde, which primarily target mucous membranes and the central nervous system ^28^. Protano et al. ^29^ have shown the relevance of such symptoms even in anatomy laboratories with concentrations within legal limits. Additionally, La Torre et al. ^2^ have highlighted the persistent exposure-related symptoms in dental schools, in which formaldehyde use remains common.

Moreover, studies such as Cammalleri et al. ^30^ underline that symptoms such as mucosal irritation may appear even when levels remain compliant. This reinforces the argument that legal thresholds may fail to fully capture all health risks.

### Biological mechanisms of formaldehyde toxicity

While this study was not designed to establish causality, the observed associations are biologically plausible and consistent with the toxicological pathways of formaldehyde. Formaldehyde, a highly reactive electrophile, readily binds to nucleophilic sites in mucosal proteins, leading to local inflammation, irritation, and oxidative stress ^31^. This explains the observed high prevalence of ocular and respiratory symptoms, even at sub-threshold concentrations. Additionally, formaldehyde can penetrate cellular membranes and induce DNA-protein crosslinks, a mechanism implicated in genotoxic and potentially carcinogenic effects ^1^. These crosslinks can interfere with DNA repair mechanisms and contribute to chromosomal instability. The disruption of neuronal function via oxidative injury or inflammation of the olfactory pathways ^23^ ^,^ ^24^ may mediate neurotoxic effects, including headaches and dizziness. Further exploring these mechanisms via biomarker-based studies would strengthen causal inferences and enhance our understanding of the dose-effect relationship in clinical environments.

Despite the well-established carcinogenic classification and general toxicological mechanisms of formaldehyde, characterizing molecular alterations in exposed healthcare workers (including oxidative stress, DNA-protein crosslinks, epigenetic modifications, and inflammatory responses) requires further research. Biomarker-based studies would help to link occupational exposure to early biological effects and explain dose-response relationships in clinical settings.

### Predictors and cumulative effects

The multivariate regression found prolonged exposure (≥ 8 hours/day, ≥ 3 days/week) as the strongest predictor of symptom clustering (AOR = 3.2; 95%CI: 1.7-5.9; p < 0.001), alongside inadequate ventilation and irregular PPE use. These findings echo those of Fan et al. ^5^, who emphasized behavioral and infrastructural factors in risk modulation.

Additionally, a positive correlation between the number of years of occupational exposure and symptom burden (Spearman’s ρ = 0.41; p < 0.01) supports a potential dose-effect relationship. Occupational studies reviewed by Scientific Committee on Occupational Exposure Limits ^26^ and in the European Chemicals Agency classification ^32^ have suggested similar findings.

The high reactivity of formaldehyde with nucleophilic sites on mucosal proteins provides a plausible mechanistic basis. According to the IARC ^1^, chronic exposure can lead to inflammation and even DNA-protein crosslinking, which may explain the progression from mild to more persistent symptoms.

### Strengths and limitations

A key strength of this study lies in its dual-methodological design, which combined quantitative environmental measurements with a structured and validated clinical symptom questionnaire. This integrated approach provided a multidimensional perspective on formaldehyde exposure and its short-term health impacts. The inclusion of a control group − comprising healthcare providers with no routinely exposure to formaldehyde − further strengthens the internal validity of the observed associations and reduces potential selection bias.

Nevertheless, several limitations require acknowledgement. Exposure assessment relied on area-based measurements rather than individual personal monitoring. Despite the placement of the JMS11 sensor near active work zones to approximate real-world exposure, this approach may fail to fully capture personal inhalation variability related to worker mobility, task-specific activities, or microenvironmental fluctuations. Consequently, this study may have misclassified individual exposure levels.

An important limitation refers to the low chemical specificity of the sensor, which measures TVOCs rather than formaldehyde alone. In clinical settings with multiple coexisting volatiles, this may result in exposure overestimation or misattribution. Accordingly, this research conservatively interpreted TVOC-based estimates, mainly using them for relative comparisons between units rather than absolute quantification.

Moreover, this study used manually triggered measurements rather than continuously automated ones, which may have missed short-term concentration peaks despite standardized hourly sampling. Despite these constraints, the JMS11 provided an ISO-compliant proxy in formaldehyde-dominant clinical settings, in which total volatile organic compounds were expected to be predominantly influenced by intentional formaldehyde use. This approach enabled feasible real-time exposure assessment in a resource-limited healthcare context.

This research stratified exposure based on reported exposure frequency and departmental assignment. It interpreted environmental concentrations against the applicable OEL, which only served as a regulatory benchmark rather than as a biological threshold or classification criterion.

Symptom assessment relied on self-reported data, which may have introduced recall bias and subjective interpretation. This study ignored objective clinical validation and biological exposure or early-effect biomarkers, limiting internal dose assessment and weakening causal inferences. Also, nonspecific reported symptoms may reflect multiple determinants beyond formaldehyde exposure alone, including disinfectants, cleaning agents, anesthetic gases, ambient particulate matter, and psychosocial stressors.

Although geographically limited to a Moroccan province, the inclusion of 12 heterogeneous clinical and laboratory units enhances the ecological validity of this research. Finally, cross-sectional studies can neither exclude residual confounding nor establish causal relationships.

Collectively, these methodological limitations may compromise the validity of exposure estimates and restrict causal interpretation, thus requiring careful consideration from evaluations of the findings in this study.

### Implications for practice and policy

The findings in this study underscore the need to strengthen and modernize formaldehyde risk management strategies in healthcare. In accordance with the hierarchy of occupational hazard control, preventive measures should prioritize the substitution of formaldehyde-based products with safer alternatives wherever feasible, followed by strengthening collective environmental controls such as ventilation upgrades, local exhaust systems, and engineering measures. Administrative controls − including exposure time reduction, staff rotation, safety training, and medical surveillance − should complement these strategies. Personal protective equipment should be considered a secondary safeguard rather than a primary control measure as it is unable to eliminate hazards at their source and relies on consistent and correct use.

These findings have direct implications for occupational health policy in LMICs, the structural and financial constraints of which often limit effective chemical risk mitigation. In resource-constrained settings, cost-effective interventions such as portable HEPA-filter units, natural cross-ventilation, and basic fume hoods may substantially reduce airborne formaldehyde levels ^6^. Training programs to improve PPE compliance have showed effectiveness across diverse contexts ^33^. In parallel, occupational health authorities increasingly encourage substitution with safer alternatives ^2^. The integrated exposure-monitoring and clinical assessment model in this study is in line with scalable frameworks for LMIC implementation ^6^.

In addition to quantitative monitoring, qualitative exposure assessment approaches may represent practical alternatives where advanced measurement infrastructure is unavailable. Structured observational checklists, task-based exposure matrices, inventory analysis of formaldehyde-containing products, ventilation assessments, and worker interviews can support preliminary risk identification and the prioritization of high-risk areas despite lower precision than quantitative measurements.

## Conclusion

This study provides robust evidence of a statistically significant association between occupational exposure to formaldehyde and the prevalence of acute symptoms in healthcare workers − even at concentrations below national occupational exposure thresholds. By integrating real-time environmental monitoring with structured clinical symptom assessment, our findings delineate a time- and dose-dependent symptom profile that is consistent with the well-documented toxicological properties of formaldehyde.

Although its cross-sectional design precludes definitive causal inference, the strength and consistency of the associations in this study − particularly in individuals with prolonged exposure, poor ventilation, and inconsistent PPE use − highlight the need for the urgent re-evaluation of current risk mitigation strategies in clinical and dental environments.

These findings align themselves with growing international concern regarding the adequacy of current regulatory thresholds and support ongoing discussions on revising occupational exposure limits toward more health-protective values, particularly for irritant and carcinogenic agents such as formaldehyde.

In LMICs such as Morocco, where infrastructural and regulatory constraints may delay the implementation of occupational health safeguards, this study shows the feasibility of integrated exposure-surveillance approaches and provides a scalable model for similar healthcare systems across North Africa and beyond.

Future research should prioritize longitudinal designs to explain causal pathways, incorporate biomarkers of exposure and early biological effects (e.g., urinary formic acid, DNA-protein crosslinks), and consider co-exposures to multiple indoor air contaminants. Such efforts will be essential for refining risk assessment frameworks, guiding evidence-based occupational health policy, and finding early molecular alterations in exposed workers, reinforcing the need to replace formaldehyde with lower-risk alternatives wherever feasible.

## Data Availability

The research data are available upon request to the corresponding author.
